# Mapping the genetic diversity of HLA haplotypes in the Japanese populations

**DOI:** 10.1038/srep17855

**Published:** 2015-12-09

**Authors:** Woei-Yuh Saw, Xuanyao Liu, Chiea-Chuen Khor, Fumihiko Takeuchi, Tomohiro Katsuya, Ryosuke Kimura, Toru Nabika, Takayoshi Ohkubo, Yasuharu Tabara, Ken Yamamoto, Mitsuhiro Yokota, Japanese Genome Variation Consortium, Koichi Akiyama, Hiroyuki Asano, Kei Asayama, Toshikazu Haga, Azusa Hara, Takuo Hirose, Miki Hosaka, Sahoko Ichihara, Yutaka Imai, Ryusuke Inoue, Aya Ishiguro, Minoru Isomura, Masato Isono, Kei Kamide, Norihiro Kato, Tomohiro Katsuya, Masahiro Kikuya, Katsuhiko Kohara, Tatsuaki Matsubara, Ayako Matsuda, Hirohito Metoki, Tetsuro Miki, Keiko Murakami, Toru Nabika, Masahiro Nakatochi, Toshio Ogihara, Keizo Ohnaka, Takayoshi Ohkubo, Hiromi Rakugi, Michihiro Satoh, Kunihiro Shiwaku, Ken Sugimoto, Yasuharu Tabara, Yoichi Takami, Ryoichi Takayanagi, Fumihiko Takeuchi, Megumi Tsubota-Utsugi, Ken Yamamoto, Koichi Yamamoto, Masayuki Yamasaki, Daisaku Yasui, Mitsuhiro Yokota, Yik-Ying Teo, Norihiro Kato

**Affiliations:** 1 Saw Swee Hock School of Public Health, National University of Singapore, Singapore 117549; 2 Life Sciences Institute, National University of Singapore, Singapore 117456; 3 NUS Graduate School for Integrative Science and Engineering, National University of Singapore, Singapore 117456; 4 Genome Institute of Singapore, Agency for Science, Technology and Research, Singapore 138672; 5 Department of Gene Diagnostics and Therapeutics, National Center for Global Health and Medicine, Tokyo, Japan 162-8655; 6 Department of Clinical Gene Therapy, Osaka University Graduate School of Medicine, Suita, Japan 565-0871; 7 Department of Human Biology and Anatomy, Graduate School of Medicine, University of the Ryukyus, Nishihara-cho, Japan 903-0215; 8 Department of Functional Pathology, Shimane University School of Medicine, Izumo, Japan 693-8501; 9 Department of Hygiene and Public Health, Teikyo University School of Medicine, Tokyo, Japan 162-8655; 10 Center for Genomic Medicine, Kyoto University Graduate School of Medicine, Kyoto, Japan 606-8501; 11 Department of Medical Chemistry, Kurume University School of Medicine, Kurume, Japan 830-0011; 12 Department of Genome Science, School of Dentistry, Aichi Gakuin University, Nagoya, Japan 464-8651; 13 Department of Statistics and Applied Probability, National University of Singapore, Singapore; 14 Department of Gene Diagnostics and Therapeutics, Research Institute, National Center for Global Health and Medicine, Tokyo, Japan.; 15 Department of internal medicine, Iwakura Hospital, Iwakura, Japan.; 16 Department of Hygiene and Public Health, Teikyo University School of Medicine, Sendai, Japan.; 17 Department of Planning of Drug Development and Clinical Evaluation, Tohoku University Graduate School of Pharmaceutical Sciences, Sendai, Japan.; 18 Studies Coordinating Centre, Research Unit Hypertension and Cardiovascular Epidemiology, KU Leuven Department of Cardiovascular Sciences, University of Leuven, Leuven, Belgium.; 19 Center for Interdisciplinary Research in Biology, College de France, Paris, France.; 20 Graduate School of Regional Innovation Studies, Mie University, Tsu, Japan.; 21 Medical Informatics Center, Tohoku University Hospital, Sendai, Japan.; 22 Department of Hygiene and Public Health, Teikyo University School of Medicine, Tokyo, Japan.; 23 Department of Functional Pathology, Shimane University School of Medicine, Izumo, Japan.; 24 Health Promotion System Science, Graduate School of Medicine, Osaka University, Suita, Japan.; 25 Department of Clinical Gene Therapy, Osaka University Graduate School of Medicine, Suita, Japan.; 26 Department of Preventive Medicine and Epidemiology, Tohoku Medical Megabank Organization, Tohoku University, Sendai, Japan.; 27 Department of Geriatric Medicine, Ehime University Graduate School of Medicine, Toon, Japan.; 28 Department of Internal Medicine, School of Dentistry, Aichi Gakuin University, Nagoya, Japan.; 29 Department of Community Medical Supports, Tohoku Medical Megabank Organization, Tohoku University, Sendai, Japan.; 30 Center for Genomic Medicine, Kyoto University Graduate School of Medicine, Kyoto, Japan.; 31 Department of Geriatric Medicine, Ehime University Graduate School of Medicine, Toon, Japan.; 32 Bioinformatics section, Center for Advanced Medicine and Clinical Research, Nagoya University Hospital, Nagoya, Japan.; 33 Department of Geriatric Medicine and Nephrology, Osaka University Graduate School of Medicine, Suita, Japan.; 34 Department of Geriatric Medicine, Graduate School of Medical Sciences, Kyushu University, Fukuoka, Japan.; 35 Department of Pharmacy, Tohoku University Hospital, Sendai, Japan.; 36 Department of Environmental and Preventive Medicine, Shimane University School of Medicine, Izumo, Japan.; 37 Department of Medicine and Bioregulatory Science, Graduate School of Medical Sciences, Kyushu University, Fukuoka, Japan.; 38 Center for International Collaboration and Partnership, National Institute of Health and Nutrition, Tokyo, Japan.; 39 Department of Medical Chemistry, Kurume University School of Medicine, Kurume, Japan.; 40 Medical Clinic of Foreign Ministry, Ministry of Foreign Affairs of Japan, Tokyo, Japan.; 41 Department of Genome Science, School of Dentistry, Aichi Gakuin University, Nagoya, Japan.

## Abstract

Japan has often been viewed as an Asian country that possesses a genetically homogenous community. The basis for partitioning the country into prefectures has largely been geographical, although cultural and linguistic differences still exist between some of the districts/prefectures, especially between Okinawa and the mainland prefectures. The Major Histocompatibility Complex (MHC) region has consistently emerged as the most polymorphic region in the human genome, harbouring numerous biologically important variants; nevertheless the presence of population-specific long haplotypes hinders the imputation of SNPs and classical HLA alleles. Here, we examined the extent of genetic variation at the MHC between eight Japanese populations sampled from Okinawa, and six other prefectures located in or close to the mainland of Japan, specifically focusing at the haplotypes observed within each population, and what the impact of any variation has on imputation. Our results indicated that Okinawa was genetically farther to the mainland Japanese than were Gujarati Indians from Tamil Indians, while the mainland Japanese from six prefectures were more homogeneous than between northern and southern Han Chinese. The distribution of haplotypes across Japan was similar, although imputation was most accurate for Okinawa and several mainland prefectures when population-specific panels were used as reference.

The advent of high-throughput genotyping technology has considerably advanced our understanding of human genetic variation globally. Landmark studies such as the Human Genome Diversity Project^1^, the International HapMap Project^2^,^3^,^4^, the Singapore Genome Variation Project^5^ and more recently the African Genome Variation Project^6^,^7^ have unveiled unprecedented insights into the genetic diversity of global populations^8^,^9^,^10^,^11^,^12^,^13^, as well as facilitated our understanding of human evolution and adaptation^14^,^15^,^16^,^17^. The Major Histocompatibility Complex (MHC) region on the short arm of chromosome 6 has consistently emerged in all of these studies as the most polymorphic region in the human genome. Spanning about 4 Mb, the MHC contains over 160 genes including the human leukocyte antigen (HLA) Class I and Class II genes, thus allowing this genomic region to play a central role in regulating the human immune system as well as in determining the compatibility of organ transplants, susceptibility to infectious and autoimmune disorders and adverse reactions to pharmacologic agents^18^,^19^,^20^,^21^,^22^.

Studies have reported considerable genetic differences at the MHC between global populations^18^,^23^, although this was similarly observed between seemingly homogeneous populations, such as the northern and southern Han Chinese^24^, and the Hondo and Ryukyu Japanese^25^. In the latter study by Yamaguchi-Kabata and colleagues, SNPs located in the HLA region were found to display the greatest allele frequency differences between the mainland Japanese (Hondo) and Japanese residing in the Ryukyu islands, when compared to SNPs found across the rest of the genome.

Although the classical serotyping, and the subsequent conversion to the HLA allele nomenclature, provides the most biologically relevant information, the availability of this information for global populations lags considerably as compared to the availability of SNP data^18^. This has led to clever strategies to statistically infer what the underlying HLA alleles are on the basis of SNP-level information, in a process known as HLA imputation which compares the target SNP data against a reference panel possessing both SNP and HLA allele data^18^,^26^. However, the accuracy of this imputation process has been shown to depend critically on whether the reference panel possesses populations that are genetically representative of the target population^27^,^28^, as underlying haplotype differences between the target and reference populations can distort this process of statistical copying. In the absence of a comprehensive HLA and SNP reference database, one useful indicator of how successful the imputation will be at recovering the HLA alleles is thus to survey the extent of haplotype diversity at Class I and Class II gene regions between the target SNP data and the reference database.

In this study, we investigate the extent of haplotype diversity in HLA Class I and Class II genes across eight Japanese populations, comprising Ryukyu samples from the Okinawa prefecture and seven groups of samples from six prefectures, located in or close to the mainland of Japan. To benchmark the extent of genetic diversity seen in these eight populations, we performed the same analyses between: (i) East Asian samples with ancestries originating from North and South China; and (ii) South Asian samples with ancestries originating from the Gujarat state in North India and from the Tamil-Nadu state in South India (Fig. 1). In order to measure the impact of the genetic differences present between the eight Japanese populations, we also evaluated the accuracy of SNP imputation in these populations with existing data from the HapMap panel, as a surrogate inference measure of how well the imputation can recover the HLA alleles for these eight Japanese populations.

## Materials and Methods

### Sample collection and genotyping

This study considered 1,400 Japanese individuals which is a subset of the 3,933 subjects in Asian Diversity Project (ADP), comprising 200 subjects each from seven regions (prefectures or cities): Amagasaki (in Hyogo Prefecture), Ehime, Fukuoka, Kita-nagoya (in Aichi Prefecture), Okinawa, Shimane and Tokyo. We additionally considered 85 unrelated Japanese samples from Tokyo in Phase 3 of the International HapMap Project (HapMap)^29^, which are specifically abbreviated as JPT to avoid confusing with the Tokyo samples from the ADP. For benchmarking, we considered the South Asian samples from the: (i) HapMap, comprising 85 unrelated Gujarati Indians in Houston (GIH); (ii) Singapore Genome Variation Project (SGVP), comprising 83 Tamil Indians in Singapore (INS); as well as the East Asian samples from the: (i) HapMap, comprising 84 unrelated Han Chinese in Beijing (CHB), which is reflected of ancestry from North China; and (ii) SGVP, comprising 96 Chinese in Singapore (CHS) which is reflected of Han Chinese ancestry from South China.

Four of the seven Japanese populations from the ADP (i.e., Amagasaki, Ehime, Fukuoka and Kita-nagoya) were genotyped on the Illumina Omni 2.5 M array, while samples from Shimane and Tokyo were genotyped on the Illumina HumanHap550, and samples from Okinawa were genotyped on the Illumina OmniExpress. The HapMap and SGVP samples were genotyped on both the Affymetrix SNP6.0 and the Illumina Human1M. Only SNPs with call rates greater than 95% and with no departure from Hardy-Weinberg equilibrium (defined as *P*_HWE_ > 0.05) were retained in our analysis, while all samples were used as these data have been already subject to prior quality control in previous publications^5^,^29^,^30^,^31^,^32^,^33^,^34^,^35^. Summary of the details of the population data we have used can be found in Supplementary Table 1.

### Basic information of seven Japanese population data

This study considered a total of 1,400 Japanese individuals, comprising 200 subjects each from seven regions (prefectures or cities) in Japan: Amagasaki (in Hyogo Prefecture), Ehime, Fukuoka, Kita-Nagoya (in Aichi Prefecture), Okinawa, Shimane and Tokyo, apart from 85 unrelated Japanese subjects from Tokyo in Phase 3 of the International HapMap Project, abbreviated as HapMap JPT. Blood samples were collected in the individual regions for anthropology and/or genetic epidemiology study. All participants from the different studies provided written informed consent, and the local ethics committees approved the protocols^30^,^31^,^32^,^33^,^34^,^35^. All genotyping were performed in accordance with relevant guidelines and regulations of the local institutes^30^,^31^,^32^,^33^,^34^,^35^. Apart from the Okinawa individuals, detailed information on the origin of four grandparents was not obtained for sampling criteria.

#### Amagasaki

The Amagasaki Study is an ongoing population-based cohort study of 5,743 individuals (3,435 males and 2,310 females), aged >18 years and recruited for a baseline examination between September 2002 to August 2003^29^. The protocol of this study was approved by the Ethics Committee of the International Medical Center of Japan^30^. All study subjects provided written informed consent for the participation.

#### Ehime

Participants in the Anti-aging study cohort (AASC) are middle-aged to elderly persons who were consecutive participants in the medical check-up program at Ehime University Hospital Anti-aging Center^31^. This medical check-up program is provided to general residents of Ehime Prefecture, and is specifically designed to evaluate aging-related disorders, including arteriosclerosis, cardiovascular diseases, physical function, and cognitive function. All study subjects provided informed consent and this study was approved by the ethics committee of Ehime University Graduate School of Medicine^31^.

#### Fukuoka

The Kyushu University Fukuoka Cohort Study is a community-based prospective epidemiologic cohort of 12,959 subjects, who participated in the baseline survey during the period from February 2004 to August 2007^32^. From this cohort, 12,569 subjects completed the questionnaire and also provided DNA for genotyping of SNPs to investigate lifestyle factors and genetic susceptibility of the so-called lifestyle-related diseases such as cardiovascular diseases, cancer, and diabetes mellitus. All participants provided written informed consent and this study was approved by the Ethics Committee of the Kyushu University Faculty of Medical Sciences^32^.

#### Kita-Nagoya

The Kita-Nagoya Genomic Epidemiology (KING) study (ClinicalTrials.gov identifier: NCT00262691) is an ongoing community-based prospective observational study of the genetic basis of cardiovascular disease and its risk factor^33^. The study recruited 3,975 Japanese subjects aged 50–80 years, who underwent community-based annual health checkups between May 2005 and December 2007. This study was approved by the Ethics Review Board of Nagoya University School of Medicine and all participants provided written informed consent^33^.

#### Okinawa

In the study of the Ryukyu population, only individuals, whose four grandparents were originated from the Ryukyu Islands, were included^34^. All participants provided written informed consent and this study was approved by the ethical committees at University of the Ryukyus, Showa University, and Kitasato University^34^.

#### Shimane and Tokyo

The Cardio-metabolic Genome Epidemiology (CAGE) Network is an ongoing collaborative effort to investigate genetic and environmental factors, and their interactions affecting cardiometabolic traits/disorders among Asian populations, including the Japanese, Vietnamese and Sri Lankan^35^. CAGE participants were recruited in a population-based or hospital-based setting, depending on the design of member studies. From this network, subjects were enrolled at separate sites in Japan including the Tokyo and Shimane districts. Subjects in the Shimane district are people who visited the Shimane Institute of Health Science for a health screening examination between July 2003 and March 2007. Subjects in the Tokyo district were selected from participants in the Hospital-based Cohort Study at the National Center for Global Health and Medicine (NCGM), Tokyo, to investigate lifestyle factors and genetic susceptibility for lifestyle-related diseases. All participants from these studies provided written informed consents, and the local ethics committees approved the protocols.

### Haplotype phasing

The genotype data for all the Japanese populations were phased with BEAGLE version 3.3.2^36^ to obtain the haplotype data necessary for our analysis of haplotype diversity. Although phased haplotypes for the HapMap and SGVP samples were available from the respective websites, these have been phased using PHASE and fastPHASE respectively. To avoid confounding the analyses due to the phasing algorithm used, the genotype data for the HapMap and SGVP samples were similarly phased with BEAGLE using the same settings. The analysis of haplotype diversity subsequently focused on a set of 1,607 SNPs between 25 Mb and 35 Mb on chromosome 6 (NCBI Build 37) that were present across all 12 populations studied.

### Population structure analyses with SNP-level F_ST_

To investigate the extent of allele frequency differences at each SNP between two populations, we calculated the SNP-level F_ST_ defined as the following by Rosenberg and colleagues^1^


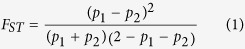


where *p*_1_ and *p*_2_ denote the frequency of a particular allele in the two populations respectively. This was calculated between every pair of populations in the collection of eight Japanese and four benchmarking populations.

### Population structure analyses with haplotype-level F_ST_

Our analyses considered six HLA genes: *HLA-A, HLA-B, HLA-C, HLA-DR, HLA-DQ* and *HLA-DP* for the purpose of measuring the extent of diversity in the observed haplotypes in the MHC region. For each of the six HLA genes, a buffer region of 100 kb up- and downstream is appended and the distinct haplotypes that are formed by the SNPs located within this extended gene region is considered. The multi-allelic version of F_ST_ in ARLEQUIN version 3.1^37^ is calculated using the observed population frequency of each haplotype to yield a haplotype-based measure of F_ST_ for each gene locus between every pair of populations. Since the samples were characterized with SNP arrays alone in the present study, the HLA haplotype data were not converted to the HLA allele nomenclature but arbitrarily numbered in the individual HLA genes.

### Population structure analyses with principal component analysis (PCA)

A series of PCAs were performed with different input. The first set of three PCAs were performed with *smartPCA* in the EIGENSOFT package^38^ using the genotype data at 240,332 SNPs present across the 12 populations, to investigate the population structure of the: (i) 12 populations; (ii) eight Japanese and two Han Chinese populations; and (iii) seven Japanese populations from the six mainland regions. The second set of three PCAs were performed with an eigen-decomposition of a *K* × *K* distance matrix, where the (*i, j*) element in the matrix is given by the average SNP-level F_ST_ between population *i* and population *j*, averaged across 1,607 common SNPs present in the interval between 25 Mb and 35 Mb on chromosome 6. The second set of three PCAs considered the same population set as the first set of three PCAs, between 12 (*K* = 12), eight (*K* = 8) and seven (*K* = 7) populations respectively. The third set of three PCAs was similar in construct to the second set, except that the analysis considered the haplotype-based F_ST_ at each of the six HLA genes.

### Evaluating imputation performance in the MHC region

We performed a series of SNP imputation in the different Japanese populations to evaluate the performance of the different population-specific and combined reference panels at the MHC. The target data to perform the imputation comprised 19 new subjects from each of the seven Japanese populations which similarly possessed the same 1,607 SNPs, although we masked 400 random SNPs and used the remaining 1,207 SNPs as input for imputation. Each population was imputed nine times, against the eight single-population reference panels and the combined East Asian reference panel which was derived from a combination of the CHB, CHS, JPT and Southeast Asian Malay samples from the SGVP or HapMap. Each of the seven population-specific panels consisted of 200 individuals, while the combined East Asian panel consisted of a modestly larger size with 350 individuals. The squared Pearson correlation coefficient (*r*^2^) between the observed genotype and the imputed allele dosage was calculated for the 400 masked SNPs across the 19 samples, and for the purpose of benchmarking imputation performance, we defined the discordance rate as 1 – *r*^2^. For benchmarking, the Han Chinese and Indian samples were similarly included in the imputation analyses, as both reference panels for the Japanese and as target data to be imputed, although the accuracy of the imputation for these samples with population-specific panels was not meaningful due to overfitting. All imputation was performed with IMPUTE version 2.3.0^39^.

## Results

### Measuring genetic distance at the MHC with SNP-level F_ST_

Between 25 Mb and 35 Mb on chromosome 6, a total of 1,607 SNPs were present in our data comprising the eight Japanese populations and the four HapMap and SGVP populations. The genetic distance between every pair of these 12 populations was measured by the average SNP-level F_ST_ values across these 1,607 SNPs. Between the eight Japanese populations, Okinawa stood out as the most distinct population, showing a minimum F_ST_ of 0.6% with Ehime and a maximum F_ST_ of 1.0% with Fukuoka, Shimane and Tokyo (Supplementary Table 1). The remaining seven Japanese populations were comparatively more homogeneous, with genetic distances in the order of 0.1% to 0.3%; the latter figure was observed in the comparison of population pairs mostly involving Ehime. The genetic distances calculated from the same 1,607 SNPs between North and South Chinese (CHB, CHS), and between North and South Indians (GIH, INS) were used to benchmark the distances seen in the Japanese populations. The distance between CHB and CHS was 0.4%, while the distance between GIH and INS was 0.5%, suggesting that the mainland Japanese populations were more homogeneous than Han Chinese from North and South China at the MHC region, whereas Okinawa was more distinct from the rest of the mainland Japanese populations than the case for genetic differences between the Gujarati and Tamil Indians.

### Principal component analyses of population structure

**Figure 2:**
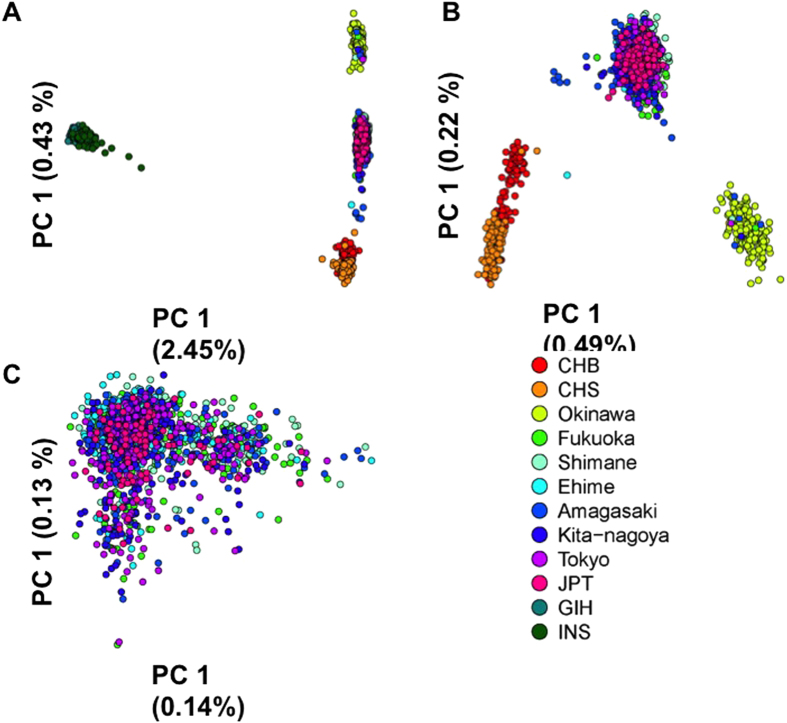
Subject-level principal component analyses with genomewide SNP data. Biplots are shown for the first two axes of variations from three different principal component analyses (PCAs) of 240,332 SNPs that are present across the genome in the eight Japanese populations and the four benchmarking populations from East and South Asia. The three different PCAs were performed on **(A)** all 12 populations; **(B)** only the eight Japanese and two Han Chinese populations; and **(C)** only the seven populations from mainland Japan. Each circle represents an individual from a particular population and is assigned a colour unique to that population that is represented in the legend on the bottom right panel.

In a preliminary PCA of 1,833 samples with genomewide data across 240,332 common SNPs in the eight Japanese and four benchmarking populations, it was evident that the two South Asian populations (GIH, INS) were significantly distinct from the East Asian populations (CHB, CHS, JPT, seven Japanese populations), although it was also clear that there were three genetic sub-clusters that corresponded to the Okinawa samples, Han Chinese and mainland Japanese respectively (Fig. 2A). The Okinawa samples were clearly distinguished from the Han Chinese and mainland Japanese samples in a manner that did not suggest that the Okinawa samples were admixed between the mainland Japanese and the Han Chinese (Fig. 2A,B), as the Okinawa samples were found in the opposite spectrum to the Han Chinese in the respective principal components. This is in good agreement with a number of findings in the history of human populations in the Japanese Archipelago; i.e., a dual structure model on the Japanese Archipelago populations^40^. In the PCA of 1,285 mainland Japanese, however, there was no evidence of any observable sub-structures between the seven populations in the analysis of genomewide data (Fig. 2C).

**Figure 3:**
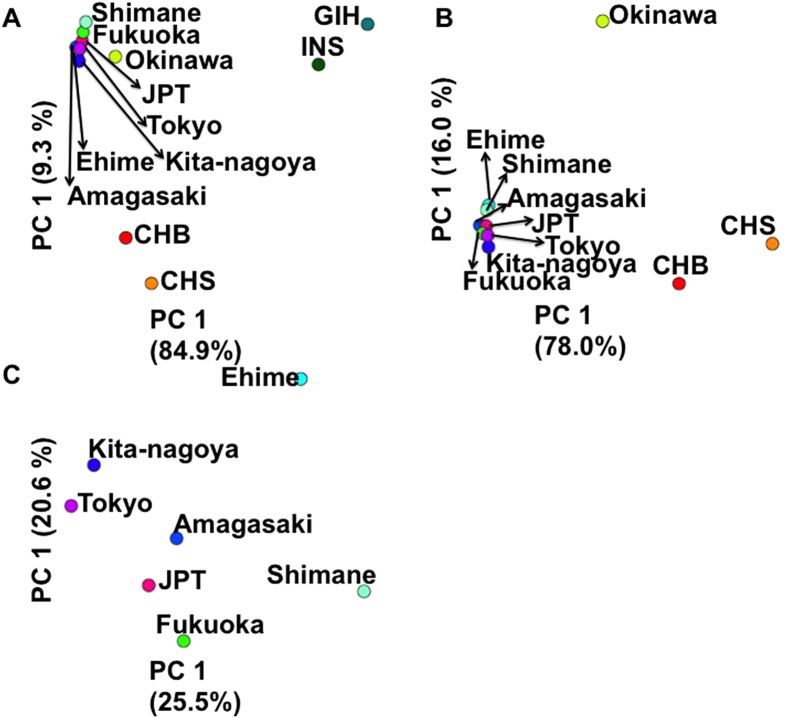
Population-level principal component analyses with SNPs at the MHC. Biplots are shown for the first two axes of variations from eigen-decompositions of distance matrices that were calculated from the average F_ST_ values between pairs of populations across 1,607 SNPs found in the interval between 25Mb and 35Mb of chromosome 6 in the eight Japanese populations and the four benchmarking populations from East and South Asia. Three different analyses were performed, involving **(A)** all 12 populations; **(B)** only the eight Japanese and two Han Chinese populations; and **(C)** only the seven populations from mainland Japan. Each circle represents a particular population and is colored with the same unique colour for that population as represented in the legend on Fig. 2.

We also performed a series of population-level PCAs using the *K × K* distance matrices (*K* represents the number of populations) constructed from the 1,607 SNPs in the 10 Mb region on chromosome 6 (see **Materials and Methods** for details). This effectively represented the genetic distance using the F_ST_ metric to quantify the extent of allele frequency differences between pairs of populations. These analyses similarly distinguished the South Asians and Han Chinese from the Japanese samples (Fig. 3A,B), as well as the Okinawa samples from the mainland Japanese samples (Fig. 3B), but appeared to provide greater resolution to the genetic differences within the seven mainland Japanese populations where Ehime and Shimane appeared to be more distinct from the remaining five populations (Fig. 3C). These observations were remarkably concordant with what we saw for the genomewide data, especially when we summarized the observations in Fig. 2 by averaging the sample-level principal component coordinates in each population to yield a single population-level coordinates for that population (Supplementary Figure 1). To further investigate the observed distinction between Ehime and Shimane and the remaining mainland Japanese populations, we pooled the F_ST_ values calculated for the 1,607 SNPs across all possible pairs of the seven mainland Japanese populations to produce an overall F_ST_ distribution. By identifying the F_ST_ values in the top 1%, we observed that there was a significant over-representation from population-pairs involving Ehime (*P*_Binomial_ = 0.0011) and Shimane (*P*_Binomial_ = 1.38 × 10^−15^). The distinction between Ehime and Shimane and the rest of the mainland Japanese samples was similarly observed in the haplotype-based PCAs at the six HLA genes (Supplementary Figure 2). Notably, the genetic differences within the seven mainland Japanese populations appeared to be more pronounced at Class II gene regions (HLA-DR, -DQ and –DP) than Class I gene regions (HLA-A, -B and -C) (Supplementary Figure 2).

### Haplotype differences between populations

Haplotypes for the 1,607 SNPs were obtained by phasing the genotype data for the 12 populations with BEAGLE. This allowed us to examine the distribution of the major haplotypes at each of the six HLA genes in each of these populations (Table 1). The definition for major haplotypes is quite arbitrary. In our study, for *HLA-A, HLA-B, HLA-C, HLA-DR*, we defined a major haplotype as possessing a population frequency of at least 10% in any of the 12 populations. While, for *HLA-DQ* and *HLA-DP*, we defined a major haplotype as possessing a population frequency of at least 6% in any of the 12 populations. This is due to the large number of haplotypes found across a larger set of SNPs at *HLA-DQ* and *HLA-DP*.

**Figure 4:**
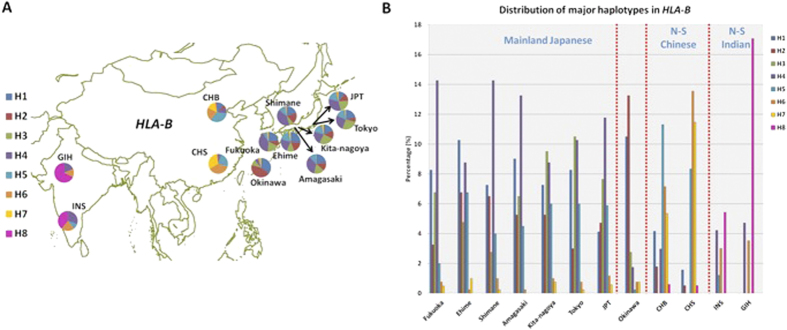
Distribution of major haplotypes at *HLA-B.* Distribution of major haplotypes found across the eight Japanese populations and the four benchmarking populations from East and South Asia at *HLA-B*, where the frequencies are illustrated **(A)** in piecharts according to the expected geographical locations, which correspond to the ancestries of the respective populations; **(B)** in barcharts to indicate the percentages for each of the major haplotypes in the 12 populations. Eight major haplotypes were observed at *HLA-B,* out of 373 unique haplotypes formed by 74 SNPs. The distribution of the major haplotypes in each of the piechart does not indicate the total sum of the frequency of the haplotypes, as label “others” was not included. The figure map was created using the R package “maps”^50^ and “mapdata”^51^ in R^52^ software.

**Figure 5:**
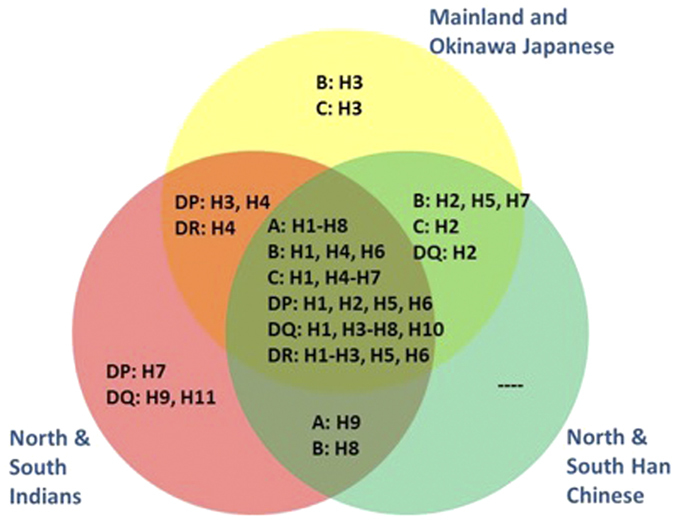
Distribution of major haplotypes across the three main ancestry groups. The eight Japanese populations and the four benchmarking populations from East and South Asia were categorised into three main ancestry groups, corresponding to the Japanese, the South Asian Indians and the East Asian Chinese. The major haplotypes observed across the six HLA genes were represented in the Venn diagram to illustrate whether they were present in each ancestry group, defined as exhibiting a non-zero frequency in at least one of the populations in the ancestry group.

Unsurprisingly, there were ancestry-specific haplotypes that were found only in South Asians or in East Asians, and the majority of the major haplotypes in Japan were shared across the different Japanese populations except that the haplotype frequencies varied between the populations to some extent (Fig. 4, Supplementary Figures 3–7). For example, in the case of *HLA-B*, although there were 373 distinct haplotypes from 74 SNPs at this locus, there were only eight major haplotypes in the 12 populations. Five of the eight major haplotypes were absent in South Asian populations (H1, H2, H3, H4, H7), while H8 was not found in any of the eight Japanese populations (Fig. 4A). One of the haplotypes (H3) appeared to be unique to the Japanese populations, and we observed that the frequency of H4 varied from 1.7% in Okinawa to 14.2% in both Fukuoka and Shimane (Fig. 4B). However, it should be noted that majority of the major haplotypes found in the HLA genes were present in all the Japanese populations and were in common with the other East and/or South Asian populations used for benchmarking (Fig. 5).

As our analysis of haplotype diversity considered mutually-distinct haplotypes that are found within a genomic region in each population, it is useful to measure to which extent these distinct haplotypes are assumed to differ. By calculating the percentage of SNP sites which differed between any two haplotypes at a locus, we observed that the majority of the major haplotypes found at the HLA loci were substantially different to each other at the level of SNPs forming individual haplotypes except at *HLA-A* where there were four major haplotypes that differed by only a single SNP (Table 2). **Imputation performance in the MHC region with different reference panels.**

An immediate consequence of haplotype variations between different Japanese populations is the impact on imputation accuracy. We investigated this in two manners: firstly, whether the accuracy changed when different single-population panels were used to impute SNP data for each Japanese population; and secondly, whether the use of a combined East Asian panel, which consists of Chinese, Japanese and Malays from public databases such as the HapMap and the SGVP, will yield better performance. The different reference panels except for the combined panel were deliberately chosen to be of comparable sizes in order to avoid any confounding due to sample size, to allow for investigation of the impact of haplotype diversity. Also, to avoid over-fitting, 19 additional samples from each of the Japanese populations (except HapMap JPT) were used as the target data for imputation.

**Figure 6:**
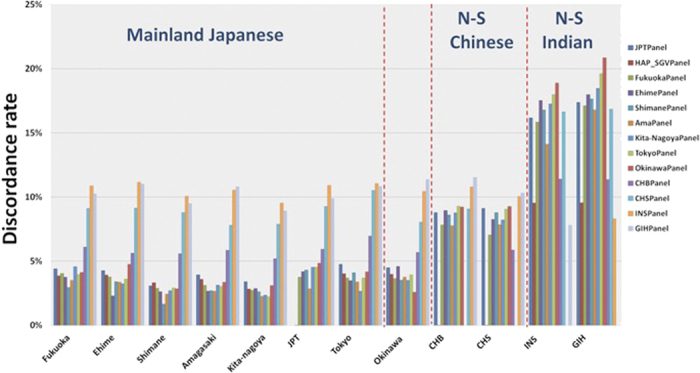
Imputation performance across the study populations. The performance of imputing samples within each of the 12 study populations was measured by the discordance rate, defined as 1 – *r*^2^, where *r*^2^ corresponds to the correlation between the observed genotype and the imputed allele dosage at 400 SNPs that were masked out of 1,607 SNPs in the MHC. For each of seven Japanese populations (except JPT), the imputation was performed on 19 additional samples which were not part of the main study and was used to construct the population-specific reference panel. On the other hand, the imputation at CHB, CHS, GIH and JPT were performed on 19 samples from the same population data, which was used to construct the reference panel and was thus subject to overfitting. The annotations of the reference panels used are as follow: JPTPanel = JPT; HAP_SGVPPanel = combined panel using the CHB, CHS, JPT samples; FukuokaPanel = Fukuoka; EhimePanel = Ehime; ShimanePanel = Shimane; AmaPanel = Amagasaki; Kita-NagoyaPanel = Kita-nagoya; TokyoPanel = Tokyo; OkinawaPanel = Okinawa; CHBPanel = CHB; CHSPanel = CHS.

We observed that the use of either the HapMap JPT panel or the combined East Asian panel yielded marginally higher discordance rates, when compared to the use of most of the single-population panels (Fig. 6, Supplementary Table 2). The latter result was surprising as the combined East Asian panel was almost double the size of the single-population panels. When imputed against single-population panels, Ehime and Okinawa samples yielded the lowest discordance rates only when the respective population-specific reference panels were used (Supplementary Table 2), providing another line of evidence to support that these two populations were more distinct from the other Japanese populations.

Three other Japanese populations (Shimane, Amagasaki, Kita-nagoya) similarly produced the lowest discordance rates when the respective population-specific reference panels were used, although this was not unique to the population-specific reference panels; there were at least one other single-population panel that yielded an equivalent level of discordance rates. For example, the lowest discordance rate of 2% was seen in Shimane when either the Shimane panel or the Amagasaki panel was used as reference. It was also evident that the use of reference panels constructed from Han Chinese or Indians yielded comparatively poorer imputation performance for the Japanese samples.

## Discussion

Our study has examined the genetic diversity between eight Japanese populations, comprising samples from Okinawa Prefecture and seven other populations samples from six prefectures, located in or close to the mainland of Japan. Our analyses focused on evaluating the genetic variation seen in the HLA Class I and Class II genes at the MHC, especially on how haplotypes differed between these populations as a surrogate to infer imputation performance for recovering the classical HLA alleles with SNP-level data. We used two pairs of populations from South Asia (GIH, INS) and East Asia (CHB, CHS) to benchmark the extent of genetic differences observed in the Japanese populations. There were multiple lines of evidence to support that Okinawa (Ryukyu) samples from Okinawa Prefecture were distinct from the mainland Japanese individuals, even more distinct than the case for genetic differences between North and South Indians. While mainland Japanese were comparatively more homogenous than Han Chinese, samples from Ehime Prefecture appeared to be marginally different from the remaining mainland Japanese individuals. The genetic differences observed between the eight Japanese populations can be partially explained by diversity at the haplotype level; the distribution of major haplotypes in each of the HLA genes has been found to vary between the populations, particularly between Okinawa and mainland Japanese populations. The haplotype variations at the MHC appear to be manifested by the discernible differences in imputation accuracy, where population-specific panels can yield marginally better performance than even a combined East Asian panel, despite the substantial homogeneity observed between the Japanese populations.

While the first phase of our imputation analysis contrasted the accuracy with the HapMap JPT reference panel against the accuracy with the population-specific and East Asian combined panels, it should be highlighted that the conventional approach to SNP imputation is to use the largest possible cosmopolitan panel, which is generally formed by a combination of samples from all available reference populations. Our intent of current genetic comparison analysis was to highlight the point that, even within the homogenous mainland Japanese populations where the predominant genetic differences were seen for the inter-population frequencies of the major haplotypes but not for the haplotype classes, there were some gains by using well-matched samples from the same populations as reference. This is important from the perspective of HLA imputation, as it has previously been shown that the use of cosmopolitan panels does not always yield superior performance to a smaller but population-specific panel^27^,^28^.

While numerous studies have thus far reported the presence of complex linkage disequilibrium (LD) patterns at the MHC^41^,^42^,^43^,^44^, they have typically focused on global populations that are unambiguously genetically distinct. In contrast, our study has investigated the haplotype differences at HLA loci between seemingly homogeneous Japanese populations, by benchmarking the observations against two pairs of non-Japanese populations from East and South Asia. One natural extension is to investigate whether there exists any LD between HLA loci, since it has been previously shown that LD in the MHC region is uncharacteristically long due to the recent positive and balancing selection^5^,^18^. To pursue this, we calculated the extent of LD between the classical HLA alleles for a set of four Asian populations in the HapMap (CHB, JPT) and SGVP (CHS, INS) (Supplementary Table 3). We observed that there were indeed long stretches of LD between the neighbouring HLA gene loci in either Class I or Class II; consequently, alleles in two neighbouring HLA genes can be found on the same haplotype in a given population (such as HLA-B*52:01 and HLA-C*12:02 in JPT), although these correlations were rarely conserved across the four populations – even within an identical ethnic group, e.g., between CHB and CHS. In this line, a previous study^45^ has reported that although there are some five-locus HLA haplotypes whose alleles exhibit strong LD, they are unique to Japanese and South Korean but not found in Chinese. Also, it has to be noted that the extent of genetic differences within the seven mainland Japanese populations is likely to be distinct between Class I and Class II gene loci even at the MHC (Supplementary Table 2). Another study^46^ has identified a recent positive selection on DPB1*04:01 in the Japanese individuals, which appears to have derived from the Korean population. Such locus-specific genetic differences in the HLA region warrant further investigation.

One may ask whether the population differentiation observed at the MHC extends to the rest of the genome, especially because in a study by Yamaguchi-Kabata and colleagues^25^, they could identify 20 regions outside the MHC that were highly differentiated between Ryukyu (Okinawa) and Hondo (mainland Japan) samples. We have examined the corresponding 20 non-MHC regions in our Japanese populations, and found similar results of genetic differentiation between the Okinawa and mainland Japanese populations in the majority of the regions with sufficient coverage of genotype data (except in two regions, see Supplementary Table 4), thus providing concordant evidence for genetic differentiation between the individuals from Okinawa Prefecture and mainland Japanese.

By virtue that the MHC is significantly more polymorphic than the rest of the genome, harbours one of the most biologically important regions in the genome and at the same time possesses long stretches of high LD, there is a need to acknowledge that broad metrics of imputation performance often calculated with input from across the genome may potentially mask important limitations with regard to imputation of SNPs in the HLA regions. Several studies^47^,^48^,^49^ have reported the risk of inaccuracies and confounding in genetic association studies in populations even with relatively small genetic differences. In this line, based on our data, we can further advocate caution in using a generic Japanese panel (e.g., JPT in the HapMap) for imputation of SNPs and HLA alleles in samples from Okinawa Prefecture.

## Additional Information

**How to cite this article**: Saw, W.-Y. *et al.* Mapping the genetic diversity of HLA haplotypes in the Japanese populations. *Sci. Rep.* **5**, 17855; doi: 10.1038/srep17855 (2015).

## Supplementary Material

Supplementary Information

## Figures and Tables

**Figure 1:**
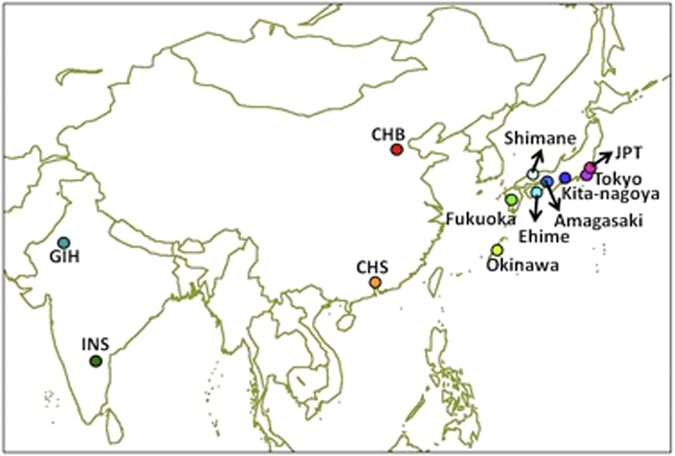
Coverage of Japanese and benchmarking populations. Illustration of the coverage of the populations tested in this study, which includes eight populations from seven prefectures in Japan, two Han Chinese populations and two South Asian populations. Each circle highlights the geographical location of a population, from which the ancestry of that population is expected to originate from. The figure map was created using the R package “maps”^50^ and “mapdata”^51^ in R^52^ software.

**Table 1:** Details of the six HLA genes (loci) in Class I (*HLA-A, HLA-B, HLA-C*) and Class II (*HLA-DR, HLA-DQ, HLA-DP*) in this study, and the number of SNPs found within each locus, which were commonly assayed across all 12 study populations.

HLA gene	Start Position	End Position	Number of SNPs	Number of Unique Haplotypes
*HLA-A*	29,810,247	30,013,868	39	110
*HLA-B*	31,221,649	31,424,989	74	373
*HLA-C*	31,136,526	31,339,913	67	377
*HLA-DR* (merging DRA1 and DRB1)	32,307,619	32,657,613	61	363
*HLA-DQ* (merging DQA1and DQB1)	32,505,183	32,734,466	70	518
*HLA-DP* (merging DPA1 and DPB1)	32,932,346	33,153,681	87	890

The genomic physical positions of each gene region are based on NCBI Build 37.

**Table 2:** The minimum dissimilarity between major haplotypes in each of the six HLA genes (loci).

Haplotype	HLA-A (n = 39)	HLA-B (n = 74)	HLA-C (n = 67)	HLA-DR (n = 61)	HLA-DQ (n = 70)	HLA-DP (n = 87)
H1	28.21	29.73	16.42	32.79	34.29	16.09
H2	2.56	2.70	17.91	32.79	21.43	11.49
H3	2.56	28.38	25.37	34.43	21.43	44.83
H4	28.21	32.43	28.36	32.79	24.29	25.29
H5	2.56	29.73	16.42	27.87	18.57	16.09
H6	28.21	2.70	17.91	27.87	8.57	11.49
H7	2.56	37.83	25.37	–	24.29	14.94
H8	35.90	18.92	–	–	24.29	–
H9	30.77	–	–	–	8.57	–
H10	–	–	–	–	18.57	–
H11	–	–	–	–	15.71	–

Dissimilarity is defined as the percentage of SNP sites where two major haplotypes found at each locus exhibited different alleles (of the SNP sites). The minimum dissimilarity for each major haplotype is then defined as the least dissimilarity observed against all other major haplotypes, thus measuring the degree of dissimilarity between the two most similar major haplotypes. A number of haplotypes were selected at each locus as follows: the haplotype frequency ≥10% for HLA-A, -B, -C, and –DR; ≥6% for HLA-DQ and –DP in any of the study populations. The number of SNPs that were assayed commonly across the 12 study populations is shown in the parenthesis at each HLA locus. For example, at HLA-A locus, the H1 haplotype shows substantial differences in alleles at ≥11 of 39 commonly-assayed SNPs ( ≥28.21%) against the other haplotypes, i.e., H2-H9 at HLA-A; H9, which is found in both Chinese and Indians but not in Japanese (see Fig. 5), is assumed to be relatively dissimilar to the other haplotypes, H1-H8, as its minimum dissimilarity is 30.77% (12 of 39SNPs).
