# Supplementary material for: Mapping the genetic diversity of HLA haplotypes in the Japanese populations
Source: Sci Rep. 2015 Dec 9;5:17855. doi: 10.1038/srep17855 (PMC4673465; doi:10.1038/srep17855)
Supplement: Supplementary Information [file srep17855-s1.doc]

**SUPPLEMENTARY MATERIAL**

**Mapping the genetic diversity of HLA haplotypes in the Japanese populations**

Woei-Yuh Saw, Xuanyao Liu, Chiea-Chuen Khor, Fumihiko Takeuchi, Tomohiro Katsuya, Ryosuke Kimura, Toru Nabika, Takayoshi Ohkubo, Yasuharu Tabara, Ken Yamamoto, Mitsuhiro Yokota, Japanese Genome Variation Consortium, Yik-Ying Teo, Norihiro Kato

**CONTENTS**

**1 Supplementary Methods 2**

1.1 R code used to draw the figure map 2

1.2 Linux command used to phase the genotypes 2

1.3 Arlequin screenshot to calculate haplotype Fst 2

1.4 Linux command used to impute genotypes in the MHC region 2

**2 Supplementary Figures 3**

- Supplementary Figure 1 3

- Supplementary Figure 2 4

- Supplementary Figure 3 5

- Supplementary Figure 4 6

- Supplementary Figure 5 7

- Supplementary Figure 6 8

- Supplementary Figure 7 9

- Supplementary Figure 8 10

- Supplementary Figure 9 11

**3 Supplementary Tables 12**

- Supplementary Table 1 12

- Supplementary Table 2 13

- Supplementary Table 3 14

- Supplementary Table 4 15

- Supplementary Table 5 16

**4 Supplementary References 17**

1. **Supplementary Methods**

1.1 R code used to draw the figure map

% library(maps)

% library(mapdata)

% map("worldHires",region=c("China","USSR","India","Japan","Bangladesh","Vietnam","Thailand","Cambodia","Myanmar","Laos","Nepal","Bhutan","Pakistan","Malaysia","North Korea","South Korea","Afghanistan","Tajikistan","Sri Lanka","Taiwan","Philippines","Mongolia"),fill=F,xlim=c(50,150),ylim=c(0,60),col="olivedrab")

1.2 Linux command used to phase the genotypes

% java –Xmx1000m –jar beagle.jar unphased=input.bgl missing=0 out=output

1.3 Arlequin screenshot to calculate haplotype Fst


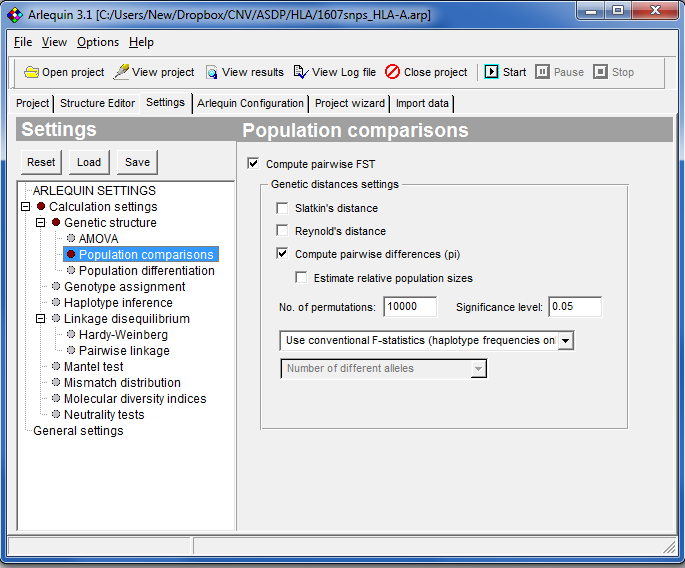


Multiallelic haplotype Fst performed by using the option of convention F-statistics with input of haplotype frequencies for each of the HLA region.

1.4 Linux command used to impute genotypes in the MHC region

% impute2 -m ref.map –h ref.hap -l ref.legend -g input_19samples.gen -allow_large_regions

-int 25e6 35e6 -Ne 20000 -o output_19samples.phased.impute2

**2 Supplementary Figures**


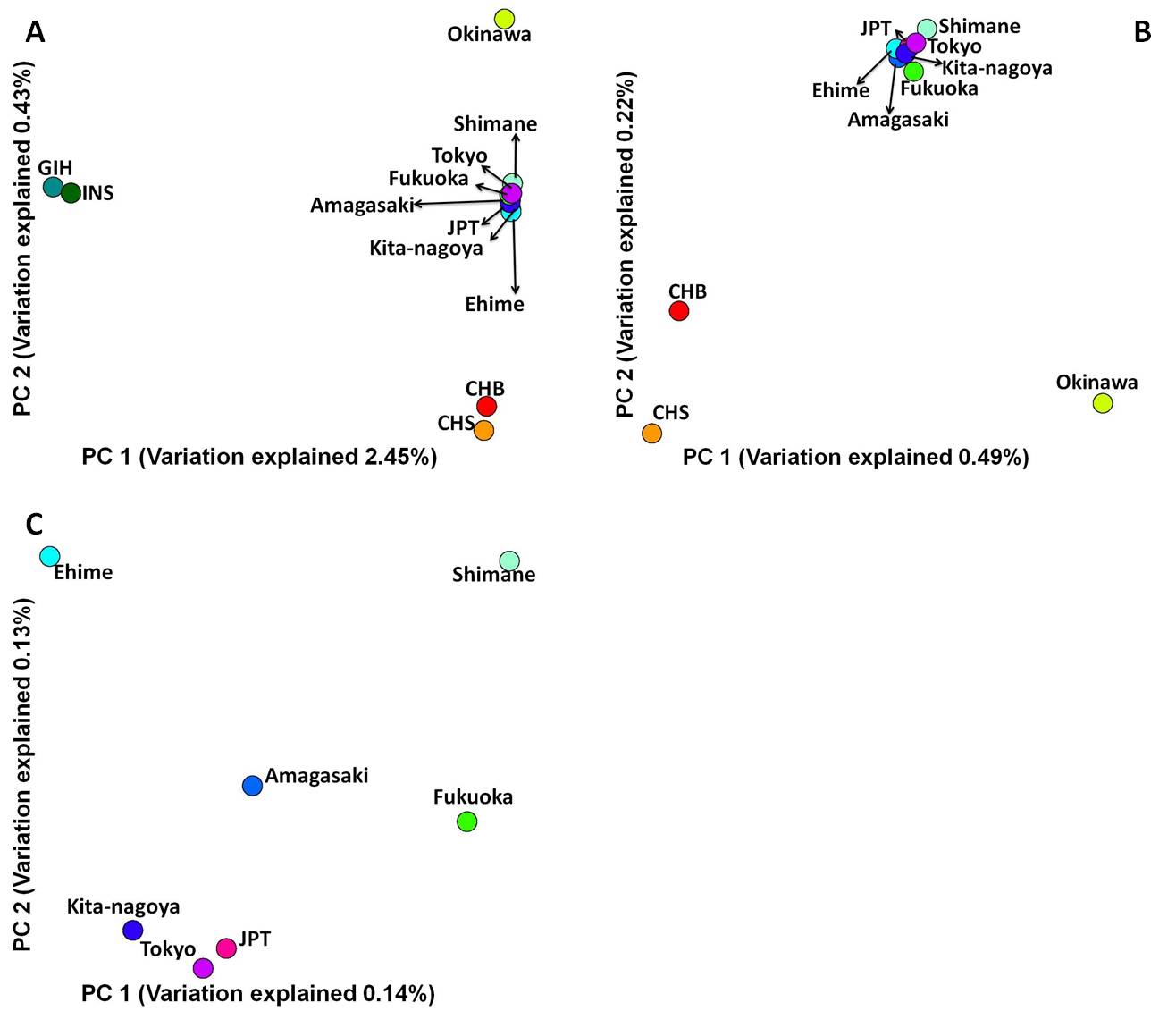


**Supplementary Figure 1.** **Population-level principal component analyses with genomewide SNPs**

These biplots are obtained from the same principal component analyses of 240,332 SNPs that are present across the genome in the eight Japanese populations and the four benchmarking populations from East and South Asia, as in **Figure 2**. The main difference is that these biplots showed the average principal component coordinates of the individuals in each population, to yield a single coordinate for the population. The three different analyses were performed on (A) all 12 populations; (B) only the eight Japanese and two Han Chinese populations; and (C) only the seven populations located in or close to the mainland of Japan. Each circle represents a particular population and is colored with the same unique color for that population, as represented in the legend on **Figure 2**.


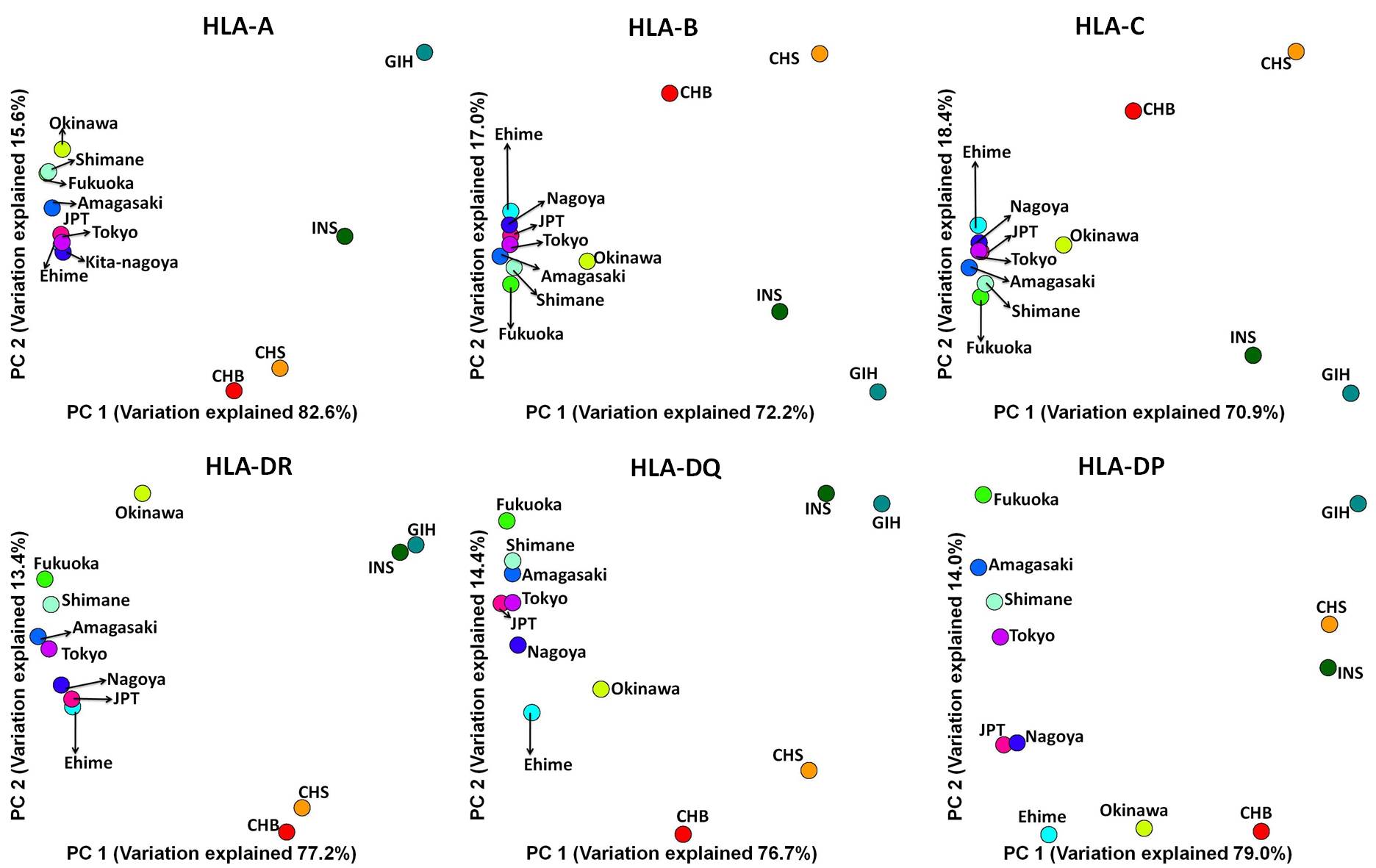


**Supplementary Figure 2. Population-level principal component analyses with SNPs at the MHC**

Biplots are shown for the first two axes of variations from eigen-decompositions of distance matrices that were calculated from the haplotype F_ST_ values between pairs of populations, across SNPs in each of the six HLA Class I and Class II genes present in the eight Japanese populations and the four benchmarking populations from East and South Asia. Each circle represents a particular population and is assigned the same unique color for that population, as represented in the legend on **Figure 2**.


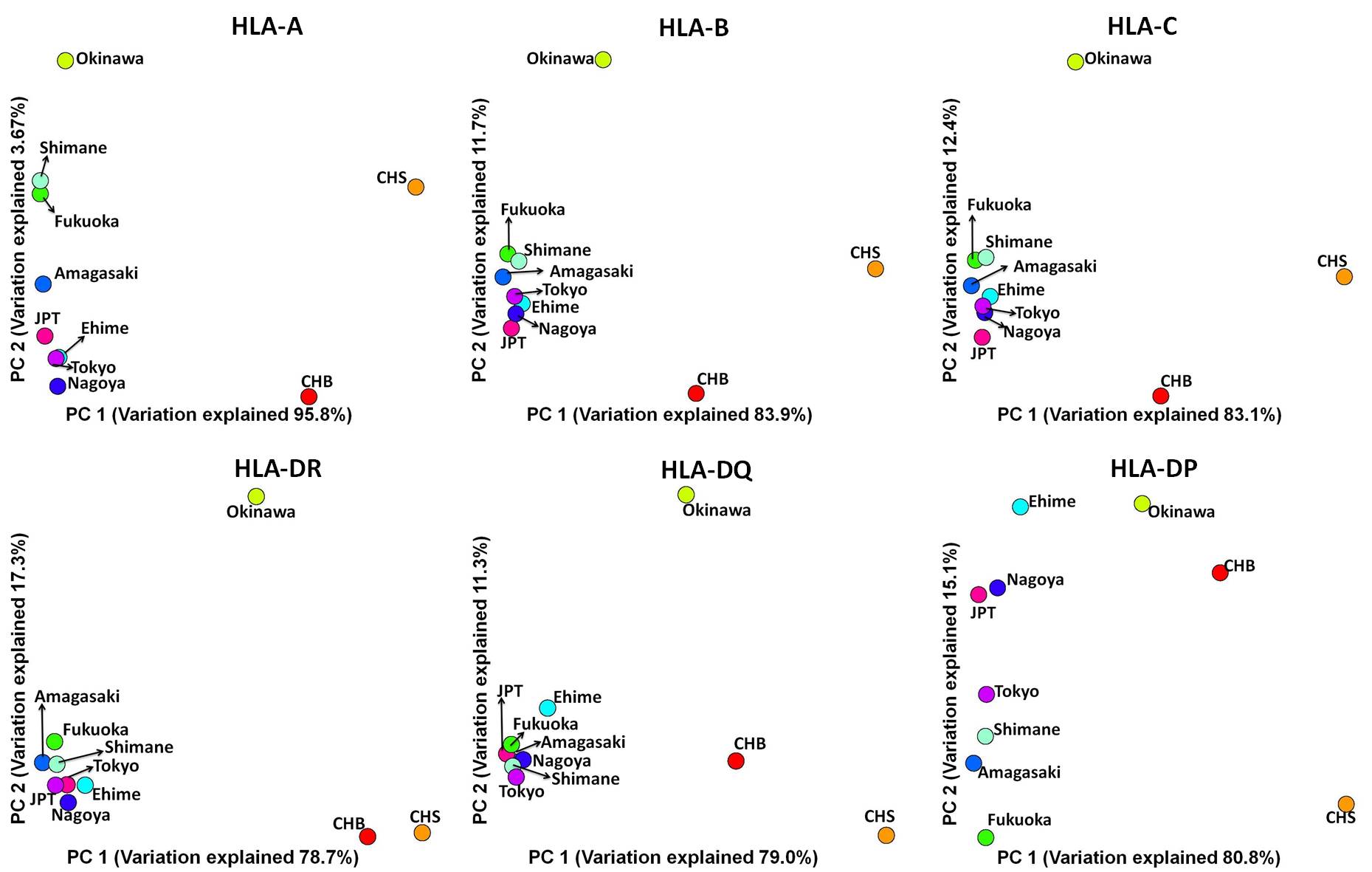


**Supplementary Figure 3. Population-level principal component analyses with SNPs at the MHC**

Biplots are shown for the first two axes of variations from eigen-decompositions of distance matrices that were calculated from the haplotype F_ST_ values between pairs of populations, across SNPs in each of the six HLA Class I and Class II genes present in the eight Japanese populations and the two benchmarking populations from East Asia. Each circle represents a particular population and is assigned the same unique color for that population, as represented in the legend on **Figure 2**.


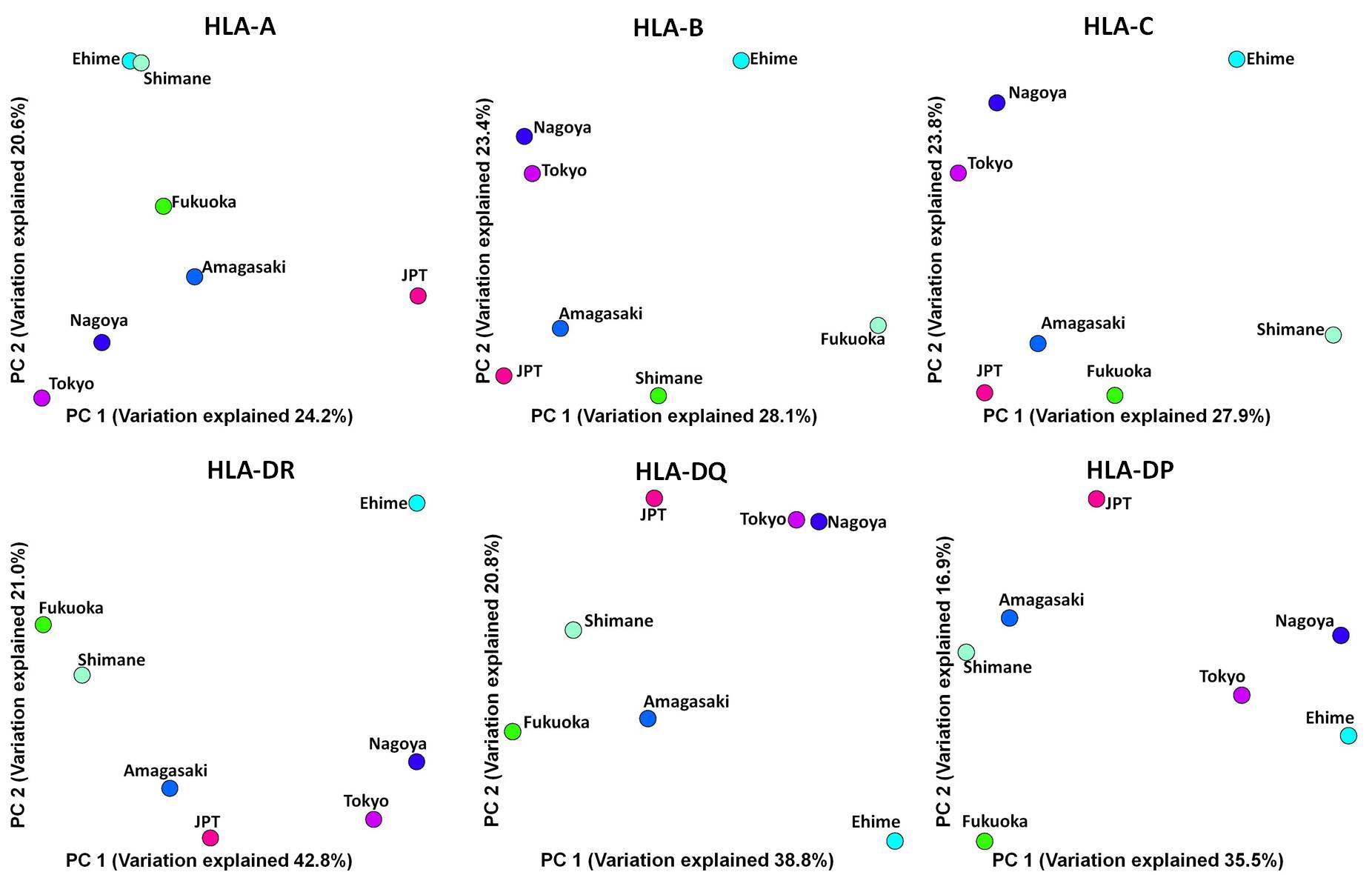


**Supplementary Figure 4. Population-level principal component analyses with SNPs at the MHC**

Biplots are shown for the first two axes of variations from eigen-decompositions of distance matrices that were calculated from the haplotype F_ST_ values between pairs of populations, across SNPs in each of the six HLA Class I and Class II genes present in the seven populations located in or close to the mainland of Japan. Each circle represents a particular population and is assigned the same unique color for that population, as represented in the legend on **Figure 2**.


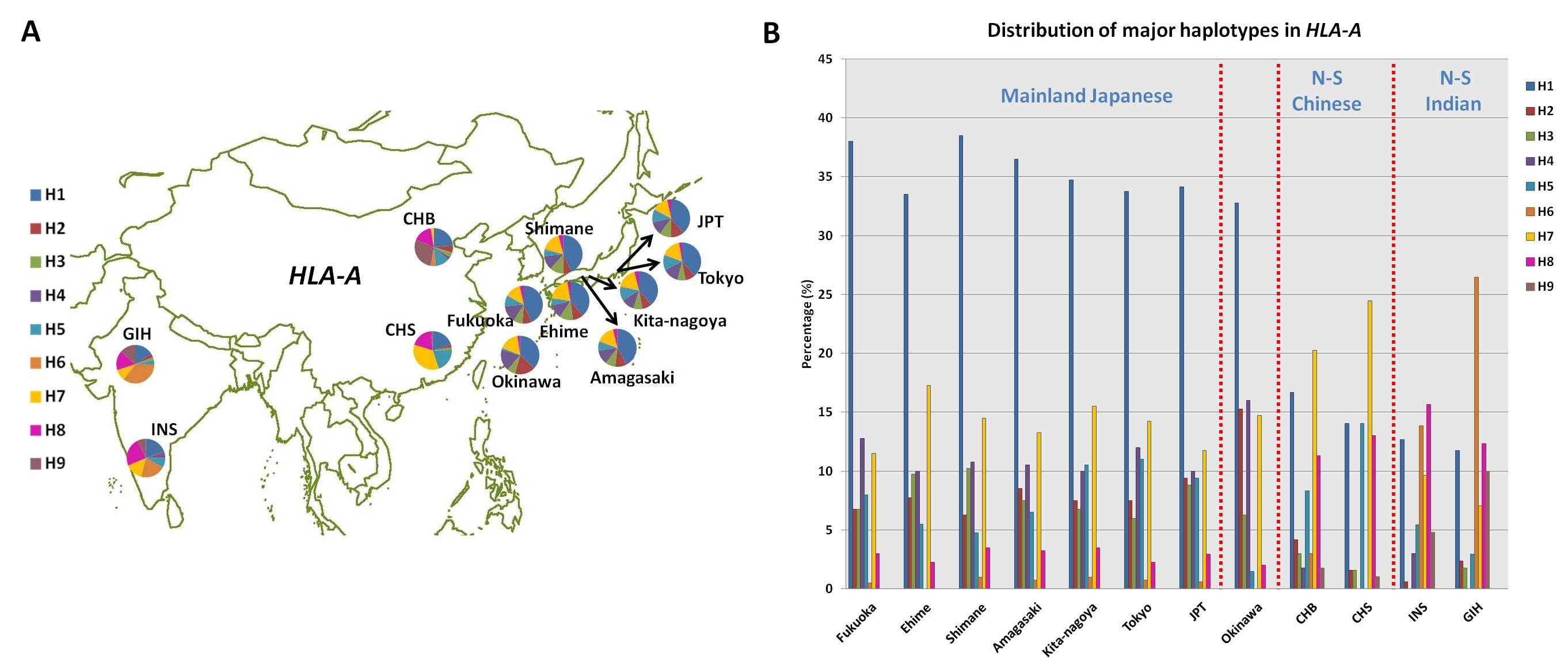


**Supplementary Figure 5. Distribution of major haplotypes at *HLA-A***

Distribution of major haplotypes found across the eight Japanese populations and the four benchmarking populations from East and South Asia at *HLA-A*, where the frequencies are illustrated (A) in piecharts according to the expected geographical locations, which correspond to the ancestries of the respective populations; (B) in barcharts to indicate the percentages for each of the major haplotypes in the 12 populations. Eight major haplotypes were observed at *HLA-A*, out of 110 unique haplotypes formed by 39 SNPs. The distribution of the major haplotypes in each of the piechart does not indicate the total sum of the frequency of the haplotypes, as label “others” was not included. The figure map was created using the R package “maps”^2^ and “mapdata”^3^ in R^4^ software.


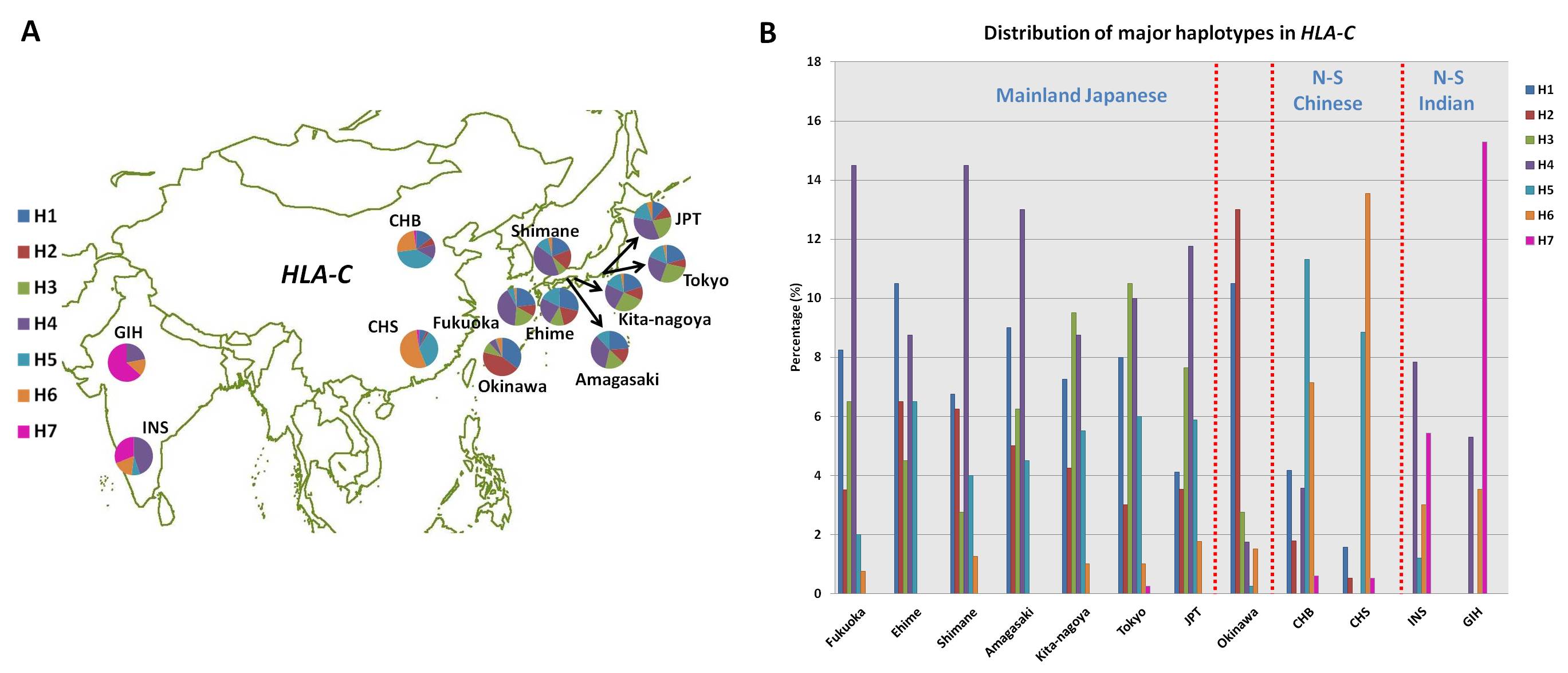


**Supplementary Figure 6. Distribution of major haplotypes at *HLA-C***

Distribution of major haplotypes found across the eight Japanese populations and the four benchmarking populations from East and South Asia at *HLA-C*, where the frequencies are illustrated (A) in piecharts according to the expected geographical locations, which correspond to the ancestries of the respective populations; (B) in barcharts to indicate the percentages for each of the major haplotypes in the 12 populations. Eight major haplotypes were observed at *HLA-C*, out of 377 unique haplotypes formed by 67 SNPs. The distribution of the major haplotypes in each of the piechart does not indicate the total sum of the frequency of the haplotypes, as label “others” was not included. The figure map was created using the R package “maps”^2^ and “mapdata”^3^ in R^4^ software.


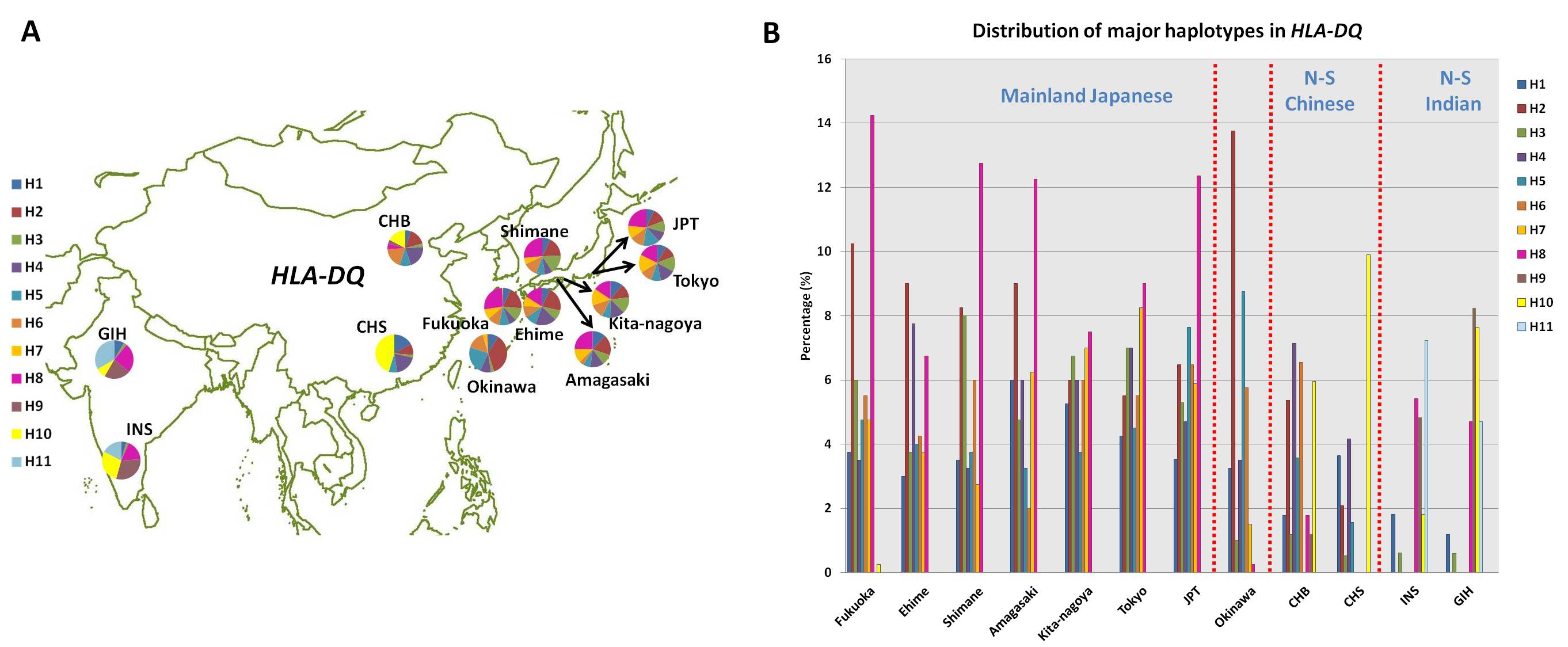


**Supplementary Figure 7. Distribution of major haplotypes at *HLA-DQ***

Distribution of major haplotypes found across the eight Japanese populations and the four benchmarking populations from East and South Asia at *HLA-DQ*, where the frequencies are illustrated (A) in piecharts according to the expected geographical locations, which correspond to the ancestries of the respective populations; (B) in barcharts to indicate the percentages for each of the major haplotypes in the 12 populations. Eight major haplotypes were observed at *HLA-DQ*, out of 518 unique haplotypes formed by 70 SNPs. The distribution of the major haplotypes in each of the piechart does not indicate the total sum of the frequency of the haplotypes, as label “others” was not included. The figure map was created using the R package “maps”^2^ and “mapdata”^3^ in R^4^ software.


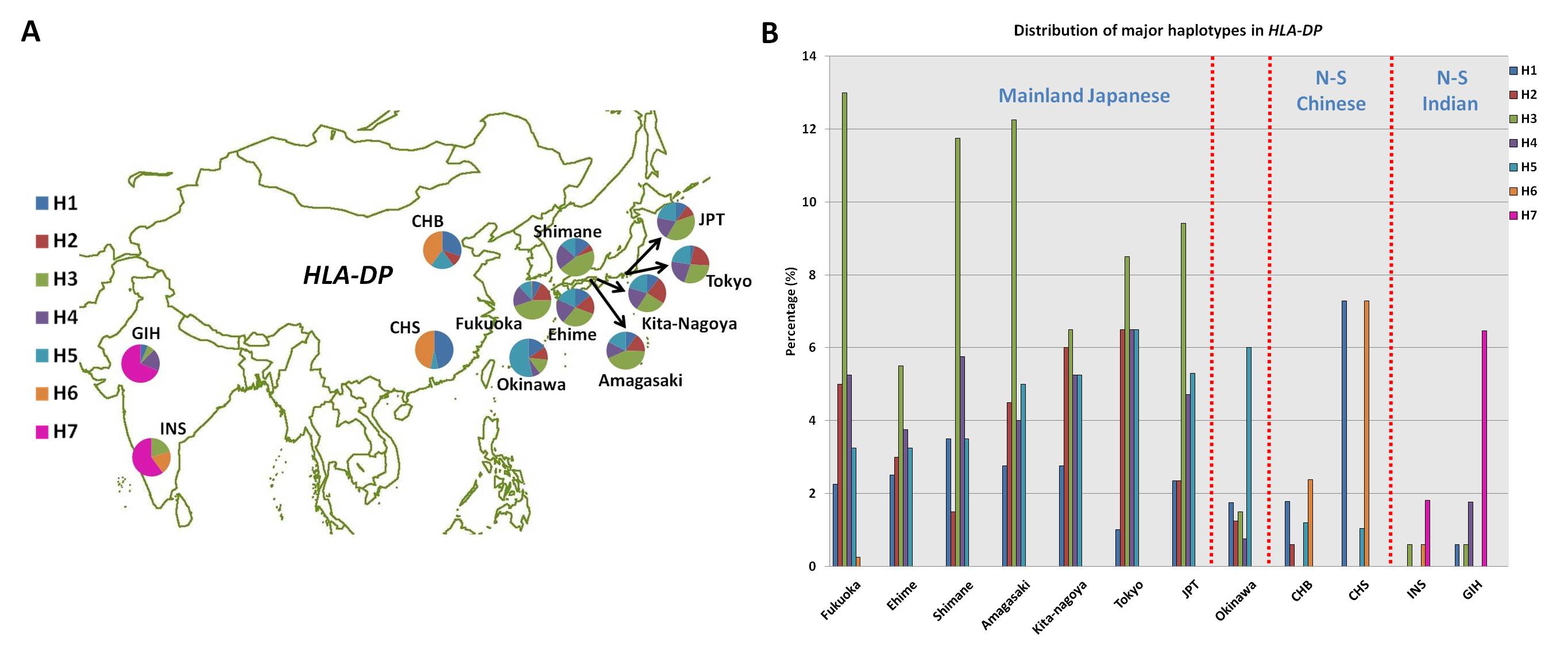


**Supplementary Figure 8. Distribution of major haplotypes at *HLA-DP***

Distribution of major haplotypes found across the eight Japanese populations and the four benchmarking populations from East and South Asia at *HLA-DP*, where the frequencies are illustrated (A) in piecharts according to the expected geographical locations, which correspond to the ancestries of the respective populations; (B) in barcharts to indicate the percentages for each of the major haplotypes in the 12 populations. Eight major haplotypes were observed at *HLA-DP*, out of 890 unique haplotypes formed by 87 SNPs. The distribution of the major haplotypes in each of the piechart does not indicate the total sum of the frequency of the haplotypes, as label “others” was not included. The figure map was created using the R package “maps”^2^ and “mapdata”^3^ in R^4^ software.

**
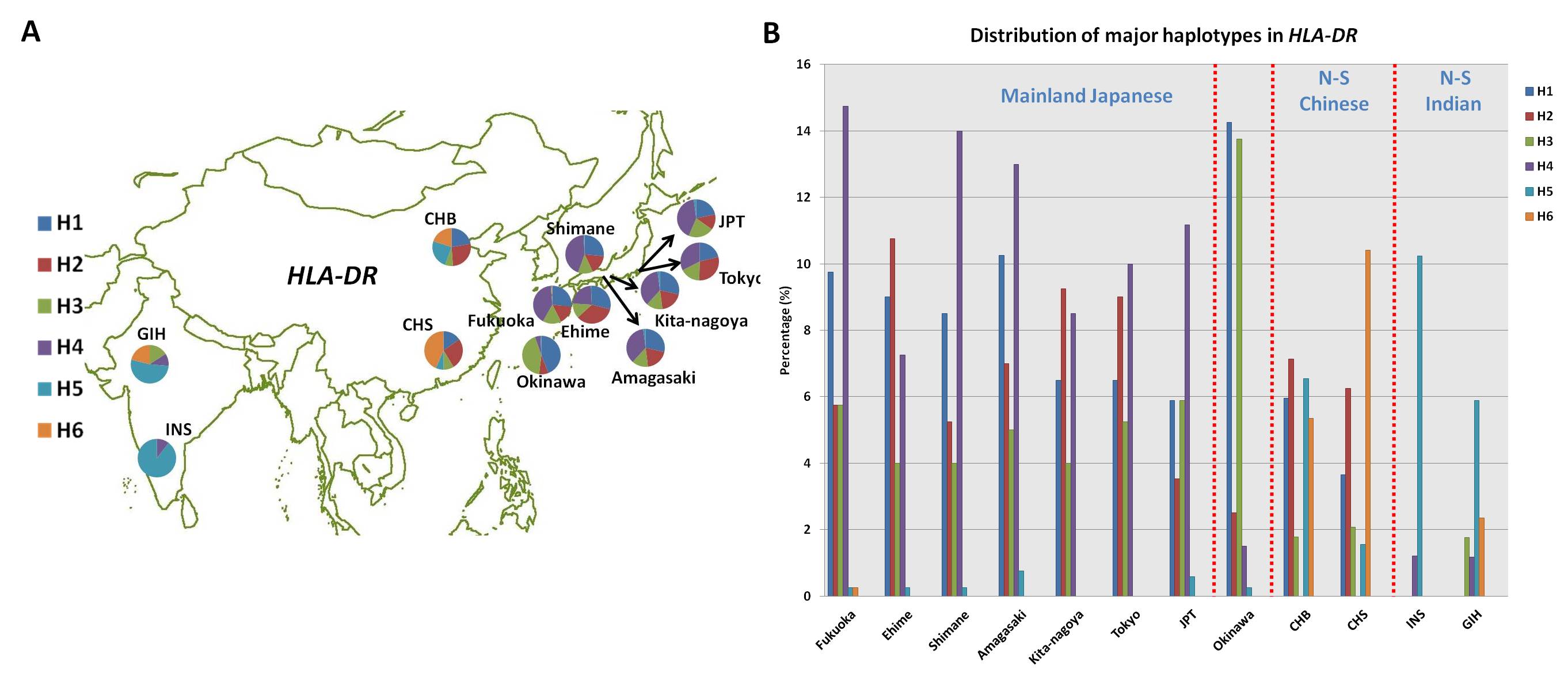
**

**Supplementary Figure 9. Distribution of major haplotypes at *HLA-DR***

Distribution of major haplotypes found across the eight Japanese populations and the four benchmarking populations from East and South Asia at *HLA-DR*, where the frequencies are illustrated (A) in piecharts according to the expected geographical locations, which correspond to the ancestries of the respective populations; (B) in barcharts to indicate the percentages for each of the major haplotypes in the 12 populations. Eight major haplotypes were observed at *HLA-DR*, out of 363 unique haplotypes formed by 61 SNPs. The distribution of the major haplotypes in each of the piechart does not indicate the total sum of the frequency of the haplotypes, as label “others” was not included. The figure map was created using the R package “maps”^2^ and “mapdata”^3^ in R^4^ software.

**3 Supplementary Tables**

**Supplementary Table 1.** Details of the population data we have used in this study and the number of SNPs found at the MHC region.

| **Dataset** | **Population** | **Number of samples** | **Genotyping platform** | **Number of SNPs at the MHC region** |
| --- | --- | --- | --- | --- |
| Japan | Amagasaki | 200 | Illumina Omni 2.5M | 18,779 |
|  | Ehime | 200 | Illumina Omni 2.5M | 18,779 |
|  | Kita-nagoya | 200 | Illumina Omni 2.5M | 18,779 |
|  | Fukuoka | 200 | Illumina Omni 2.5M | 18,779 |
|  | Shimane | 200 | Illumina HumanHap550 | 1,815 |
|  | Tokyo | 200 | Illumina HumanHap550 | 1,815 |
|  | Okinawa | 200 | Illumina OmniExpress | 7,510 |
| HapMap 3 | CHB | 84 | Affymetrix SNP6.0 + Illumina Human1M | 9,237 |
|  | JPT | 85 | Affymetrix SNP6.0 + Illumina Human1M | 9,237 |
|  | GIH | 85 | Affymetrix SNP6.0 + Illumina Human1M | 9,237 |
| SGVP | CHS | 96 | Affymetrix SNP6.0 + Illumina Human1M | 9,692 |
|  | INS | 83 | Affymetrix SNP6.0 + Illumina Human1M | 9,698 |

**Supplementary Table 2.** Average SNP-level F_ST_ between pairs of populations calculated for the 1,607 SNPs present in the MHC region.

| **SNPs** | **Okinawa** | **Ehime** | **Fukuoka** | **Kita- nagoya** | **Amagasaki** | **Shimane** | **Tokyo** | **JPT** |
| --- | --- | --- | --- | --- | --- | --- | --- | --- |
| **Ehime** | 0.006 | 0 | - | - | - | - | - | - |
| **Fukuoka** | 0.010 | 0.003 | 0 | - | - | - | - | - |
| **Kita-nagoya** | 0.009 | 0.002 | 0.002 | 0 | - | - | - | - |
| **Amagasaki** | 0.008 | 0.002 | 0.001 | 0.002 | 0 | - | - | - |
| **Shimane** | 0.010 | 0.002 | 0.002 | 0.003 | 0.002 | 0 | - | - |
| **Tokyo** | 0.010 | 0.003 | 0.002 | 0.001 | 0.002 | 0.003 | 0 | - |
| **JPT** | 0.009 | 0.003 | 0.002 | 0.002 | 0.002 | 0.002 | 0.001 | 0 |

**Supplementary Table 3.** Tabulation of the correlation between the observed genotypes and the imputed allele dosages at 400 masked SNPs, which were randomly chosen out of 1,607 SNPs that were present in the eight Japanese populations and the four benchmarking populations from East and South Asia. For seven of the Japanese populations (except JPT), imputation was performed on 19 additional samples that were not part of the reference panels in order to avoid overfitting. The cells colored in grey indicated the best performance observed for each population (across the columns) given the reference panel (across the rows).

| **Ref Panel** | **Fukuoka** | **Ehime** | **Shimane** | **Amagasaki** | **Kita-nagoya** | **Tokyo** | **Okinawa** | **JPT** | **CHB** | **CHS** | **INS** | **GIH** |
| --- | --- | --- | --- | --- | --- | --- | --- | --- | --- | --- | --- | --- |
| **JPTPanel** | 0.96 | 0.96 | 0.97 | 0.96 | 0.97 | 0.95 | 0.96 | 1.00 | 0.91 | 0.91 | 0.84 | 0.83 |
| **HAP_SGVPanel** | 0.96 | 0.96 | 0.97 | 0.96 | 0.97 | 0.96 | 0.96 | 1.00 | 1.00 | 1.00 | 0.90 | 0.90 |
| **FukuokaPanel** | 0.96 | 0.96 | 0.97 | 0.97 | 0.97 | 0.96 | 0.96 | 0.96 | 0.92 | 0.93 | 0.84 | 0.83 |
| **EhimePanel** | 0.96 | 0.98 | 0.97 | 0.97 | 0.97 | 0.97 | 0.95 | 0.96 | 0.91 | 0.92 | 0.82 | 0.82 |
| **ShimanePanel** | 0.97 | 0.97 | 0.98 | 0.97 | 0.97 | 0.96 | 0.96 | 0.96 | 0.91 | 0.91 | 0.83 | 0.82 |
| **AmaPanel** | 0.96 | 0.97 | 0.98 | 0.97 | 0.98 | 0.97 | 0.96 | 0.97 | 0.92 | 0.92 | 0.86 | 0.83 |
| **Kita-nagoyaPanel** | 0.95 | 0.97 | 0.97 | 0.97 | 0.98 | 0.97 | 0.96 | 0.95 | 0.91 | 0.92 | 0.83 | 0.82 |
| **TokyoPanel** | 0.96 | 0.96 | 0.97 | 0.97 | 0.98 | 0.96 | 0.96 | 0.95 | 0.91 | 0.91 | 0.82 | 0.80 |
| **OkinawaPanel** | 0.96 | 0.95 | 0.97 | 0.97 | 0.97 | 0.96 | 0.97 | 0.95 | 0.91 | 0.91 | 0.81 | 0.79 |
| **CHBPanel** | 0.94 | 0.94 | 0.94 | 0.94 | 0.95 | 0.93 | 0.94 | 0.94 | 1.00 | 0.94 | 0.89 | 0.89 |
| **CHSPanel** | 0.91 | 0.91 | 0.91 | 0.92 | 0.92 | 0.89 | 0.92 | 0.91 | 0.91 | 1.00 | 0.83 | 0.83 |
| **INSPanel** | 0.89 | 0.89 | 0.90 | 0.89 | 0.90 | 0.89 | 0.90 | 0.89 | 0.89 | 0.9 | 1.00 | 0.92 |
| **GIHPanel** | 0.90 | 0.89 | 0.91 | 0.89 | 0.91 | 0.89 | 0.89 | 0.90 | 0.88 | 0.9 | 0.92 | 1.00 |

**Supplementary Table 4.** Linkage disequilibrium (LD) between alleles in the neighboring HLA genes in Class I and Class II calculated from the HLA allele data for CHB, CHS, JPT and INS. The pairwise LD between each pair of the HLA alleles was calculated using the Haploxt program, part of the GOLD package ^1^, applied to the haplotypes with binary-encoded HLA alleles. The extent of LD was quantified using a genetic correlation coefficient, *r*^2^. The table shows only pairs of HLA alleles, where *r*^2^ was at least 0.80 in one of the four populations, and where the frequency of the allele was at least 5% in that population.

|  |  | **correlation coefficient, r²** | | | |
| --- | --- | --- | --- | --- | --- |
| **HLA allele** | **HLA allele** | **JPT (n=82)** | **CHB (n=86)** | **CHS (n=192)** | **INS (n=166)** |
| C*12:02 | B*52:01 | 1.00 | - | - | 0.85 |
| C*14:03 | B*44:03 | 1.00 | - | - | - |
| DRB1*09:01 | DQB1*03:03 | 0.85 | 0.94 | - | - |
| DQA1*01:03 | DQB1*06:01 | 0.78 | 0.86 | - | - |
| C*03:02 | B*58:01 | - | - | 1.00 | - |
| DRB1*040:6 | DQB1*03:02 | - | - | 0.94 | - |
| DRB1*07:01 | DQA1*02:01 | - | - | - | 0.96 |

Supplementary Table 5. The study by Yamaguchi-Kabata and colleagues identified 20 non-MHC regions that were highly differentiated between Hondo (mainland Japanese) and Ryukyu (Okinawa) clusters. The numbers illustrated in the three columns under the header “Yamaguchi-Kabata et al” were extracted from the publication by Yamaguchi-Kabata. We performed a similar calculation of haplotype F_ST_ for the Japanese populations in our study; four populations (Amagasaki, Ehime, Fukuoka and Kita-nagoya) against Okinawa. The HF_ST_ (min, max) under the header “Our study” shown below for each chromosomal region represents the minimum and maximum of the HF_ST_ from the four population pairs. The genomic physical positions of each region are based on NCBI Build 37.

|  |  |  |  |  | **Yamaguchi-Kabata**  **et al** | | | **Our study** | | |
| --- | --- | --- | --- | --- | --- | --- | --- | --- | --- | --- |
| **Region** | **Chr** | **Start** | **End** | **Gene** | **# SNPs** | **# haps** | **HF_ST_** | **# SNPs** | **# haps** | **HF_ST_  (min,max)** |
| R1 | 2 | 38486570 | 38490867 | - | 4 | 7 | 0.0276 | 4 | 9 | (0.0142,0.0227) |
| R2 | 3 | 176505197 | 176524770 | - | 4 | 4 | 0.0277 | 6 | 7 | (0.0406,0.0834) |
| R3 | 3 | 176530374 | 176556670 | - | 4 | 9 | 0.0297 | 6 | 11 | (0.0412,0.0866) |
| R4 | 3 | 187391248 | 187401981 | - | 4 | 5 | 0.0390 | 7 | 7 | (0.0203,0.0526) |
| R5 | 4 | 67733903 | 67758600 | - | 4 | 6 | 0.0251 | 4 | 4 | (0.0011,0.0044) |
| R6 | 4 | 80121894 | 80146690 | - | 4 | 3 | 0.0291 | 2 | - | - |
| R7 | 4 | 143936903 | 143972745 | LOC729675 | 4 | 3 | 0.0352 | 2 | - | - |
| R8 | 4 | 144060464 | 144086245 | LOC729675 | 4 | 4 | 0.0271 | 2 | - | - |
| R9 | 4 | 167933010 | 167972084 | SPOCK3 | 4 | 6 | 0.0288 | 6 | 9 | (0.0197,0.0361) |
| R10 | 5 | 53360059 | 53364154 | - | 4 | 2 | 0.0299 | 4 | 5 | (0.0132,0.0329) |
| R11 | 5 | 129591296 | 129616513 | - | 4 | 3 | 0.0302 | 3 | 4 | (0.0558,0.0767) |
| R12 | 6 | 31132085 | 31136453 | POU5F1 | 4 | 5 | 0.0277 | 6 | 7 | (0.0182,0.0744) |
| R13 | 7 | 146952081 | 146955688 | CNTNAP2 | 4 | 6 | 0.0304 | 3 | 7 | (0.0278,0.0599) |
| R14 | 7 | 146966876 | 146970047 | CNTNAP2 | 4 | 7 | 0.0280 | 3 | 5 | (0.0318,0.0706) |
| R15 | 7 | 146980556 | 146998293 | CNTNAP2 | 4 | 6 | 0.2650 | 2 | - | - |
| R16 | 10 | 53234928 | 53237647 | PRKG1 | 4 | 7 | 0.0300 | 3 | 6 | (0.0337,0.0528) |
| R17 | 10 | 53240022 | 53242283 | PRKG1 | 4 | 7 | 0.0272 | 3 | 8 | (0.0317,0.0468) |
| R18 | 10 | 53242459 | 53244645 | PRKG1 | 4 | 4 | 0.0338 | 3 | 5 | (0.0367,0.0658) |
| R19 | 12 | 71197248 | 71219976 | PTPRR | 4 | 4 | 0.0331 | 6 | 11 | (0.0006,0.0153) |
| R20 | 12 | 111393519 | 111410537 | - | 4 | 3 | 0.0260 | 8 | 16 | (0.0502,0.0794) |

**4 Supplementary References**

1. Abecasis, G.R. & Cookson, W.O. GOLD--graphical overview of linkage disequilibrium. Bioinformatics 16, 182-3 (2000).

2. Original S code by Richard A. Becker and Allan R. Wilks. R version by Ray Brownrigg. Enhancements by Thomas P Minka <tpminka@media.mit.edu>. maps: Draw Geographical Maps. (2014) R package version 2.3-9. <http://CRAN.R-project.org/package=maps>, Date of access: 06/11/2014

3. Original S code by Richard A. Becker and Allan R. Wilks. R version by Ray Brownrigg. mapdata: Extra Map Databases. (2014) R package version 2.2-3. <http://CRAN.R-project.org/package=mapdata>, Date of access: 06/11/2014

4. R Core Team, R: A language and environment for statistical computing. R Foundation for Statistical Computing (2013) Available at: [www.R-project.org/](http://www.r-project.org/), Date of access: 01/01/2014
